# Speckle tracking echocardiography: a reliable tool for right ventricle function evaluation in severe tricuspid regurgitation

**DOI:** 10.1186/s44348-024-00034-1

**Published:** 2024-09-26

**Authors:** Ju-Hee Lee

**Affiliations:** Division of Cardiology, Chungbuk National University Hospital, Chungbuk National University College of Medicine, Cheongju, Republic of Korea

In recent years, our understanding of the right ventricle (RV) has evolved significantly, recognizing it not merely as a conduit for blood flow but for its distinct functional significance. RV systolic dysfunction is a well-established prognostic indicator in various cardiovascular diseases, playing a crucial role in risk stratification and guiding optimal therapy [1–11]. This is especially pertinent in cases of severe tricuspid regurgitation (TR), where RV function becomes a vital prognostic factor due to the resultant volume overload and right-sided heart remodeling [12].

However, assessing RV function poses considerable challenges due to its complex triangular anatomy and distinct functional mechanisms, which differ markedly from those of the left ventricle (LV) [1, 2, 12, 13]. The RV’s crescentic shape and separation between inflow and outflow tracts preclude uniform geometric assumptions for volume measurement. Capturing the entire RV in a single view is difficult due to its distinct anatomical configuration, and defining the endocardial border is made difficult by its pronounced trabeculation. Furthermore, the RV’s location just behind the sternum complicates image acquisition. Its orientation and position can vary widely, and its function is highly susceptible to changes in preload, afterload, or left ventricular function. Additionally, the RV's contraction-relaxation dynamics are complex and differ across its various segments.

Conventional echocardiographic methods to evaluate RV function include RV fractional area change (RVFAC), tricuspid annular plane systolic excursion (TAPSE), tricuspid S’ velocity, and the RV myocardial performance index (RV Tei index) [1, 2, 13]. However, these methods reflect only longitudinal function, are angle- and load-dependent, and exhibit poor reproducibility, limiting their usefulness for serial measurements and making it difficult to compare and interpret results over time. Cardiac magnetic resonance imaging (CMR) is considered the gold standard for RV volumetric and functional assessment according to clinical guidelines and numerous studies, but its routine use is limited by its significant expense and lengthy examination process [1, 2, 13].

Unlike conventional echocardiographic parameters, strain measurements evaluate the intrinsic function of the myocardium, allowing differentiation between active and passive motion [1, 2]. As validated in both animal and human studies, RV longitudinal strain is a reliable and accurate measure of RV systolic function. It has a stronger correlation with RV systolic function and provides greater prognostic value than parameters such as TAPSE and tricuspid S’ velocity, which represent only the displacement of the RV basal segment [1, 2]. Although the utility of RV strain has been well-documented in various cardiovascular diseases including heart failure, ischemic heart disease, and pulmonary hypertension, there are relatively few studies on RV strain specifically in patients with severe TR [12].

In this issue of the *Journal of Cardiovascular Imaging*, Moon et al. [14] explore the efficacy of speckle tracking echocardiography (STE) in assessing RV systolic function in patients with severe TR. Given the difficulties in evaluating RV function using traditional echocardiography due to the RV's complex geometry and the dilation often seen in severe TR, this study provides critical insights into alternative imaging modalities that could enhance clinical practice. The authors retrospectively analyzed 87 consecutive patients with isolated severe TR who underwent both echocardiography and CMR within a 30-day window. RV free-wall longitudinal strain (RVFWLS) demonstrated the best correlation with CMR-derived RV ejection fraction (RVEF; r = –0.37, P < 0.001), surpassing RV global longitudinal strain and traditional metrics like RVFAC. Although it showed a modest correlation, this finding underscores RVFWLS's potential as a reliable surrogate for RV function in clinical settings where CMR is not feasible. Furthermore, RVFWLS exhibited superior discrimination of RV dysfunction, defined as CMR-derived RVEF < 35%, with an area under the curve of 0.801. The combined assessment of RVFWLS, indexed RV end-systolic area, and RVEF further improved the discriminatory value for RV systolic dysfunction.

It is important to consider that while CMR is considered the gold standard for evaluating RV function, it is not without its limitations. For instance, the accuracy of CMR-derived RVEF can be affected by factors such as image resolution and patient movement during the scan, which can lead to variability in the measurements​. Furthermore, the increased regurgitant volume in TR patients may result in an overestimation of RV function, making it challenging to rely solely on CMR-derived metrics [15]. Additionally, studies have identified CMR-derived RVFWLS as an independent predictor of mortality in patients with severe functional TR [16]. These limitations of CMR-derived parameters suggest that measuring RV strain using STE may be particularly valuable in severe TR patients, and its potential role in diagnosing and predicting RV dysfunction warrants further exploration.

Given the high cost and limited availability of CMR, the ability to reliably assess RV dysfunction through RV strain echocardiography is highly valuable, particularly for the ongoing monitoring and management of patients with severe TR. This approach allows for more frequent and accessible evaluations, facilitating timely clinical decisions, especially regarding the timing of surgical interventions. The use of RVFWLS as a key parameter could become standard practice, aiding in the early detection of RV systolic dysfunction and potentially improving patient outcomes.

However, several challenges should be addressed before RV strain can be widely adopted in clinical practice [2]. First, the establishment of normal reference values and cutoffs for various pathological conditions is necessary. Studies have shown that RV strain values differ by sex, age, and vendor, which must be considered when interpreting results. Additionally, further research is needed to establish pathologic cutoff values for different diseases. Intervendor variability also presents a significant challenge. Using the same echocardiographic machine and software for consecutive examinations can mitigate this variability. Another issue is the ongoing debate about whether to include the interventricular septum in RV strain measurements. Anatomically, the RV and LV share the interventricular septum, and the LV contributes significantly to RV systolic function. Current guidelines recommend measuring RVFWLS, but conflicting research results on the correlation with RV function and prognosis indicate that further studies are needed. Lastly, the lack of prospective studies on RV strain is a concern. Most existing research relies on retrospective analyses, which are susceptible to bias. Prospective studies with larger sample sizes are needed to address intervendor variability and explore the effects of this variability on treatment patterns and prognostication via RV strain.

In conclusion, this study by Moon et al. [14] significantly advances our understanding of RV function assessment in severe TR. By validating the use of STE-derived RV strain against CMR, the study highlights a practical, cost-effective alternative for routine clinical use, potentially enhancing patient care through more accessible and frequent evaluations.

## Data Availability

Not applicable.

## Abbreviations

CMR: Cardiac magnetic resonance imaging
LV: Left ventricle
RV: Right ventricle
RVEF: Right ventricle ejection fraction
RVFAC: Right ventricle fractional area change
RVFWLS: Right ventricle free-wall longitudinal strain
STE: Speckle tracking echocardiography
TAPSE: Tricuspid annular plane systolic excursion
TR: Tricuspid regurgitation

## Data Availability

No datasets were generated or analysed during the current study.
